# Supplementary material for: Non-Apoptotic Programmed Cell Death as Targets for Diabetic Retinal Neurodegeneration
Source: Pharmaceuticals (Basel). 2024 Jun 26;17(7):837. doi: 10.3390/ph17070837 (PMC11279440; doi:10.3390/ph17070837)
Supplement: Supplementary file 1 [file pharmaceuticals-17-00837-s001.zip › pharmaceuticals-3022191-supplementary.pdf]

Table S1 Non-Apoptotic Programmed Cell Death in Diverse Retinal Cell Populations of DRN

PCD	First defined	Classical signaling molecules	Pathophysiology related to DRN	Types of cells involved in DRN
Ferroptosis	Dixon et al, 2012 [22]	GPX4<br>GSH<br>system Xc-<br>PUFA<br>ACSL4<br>LPCAT3	Oxidative stress<br>Lipid peroxidation<br>I/R injury<br>Inflammation	**RPE** The primary cell type undergoing ferroptosis, both at animal and cellular levels [39-40]. Induced by oxidative stress [41-42] and regulated by non-coding RNAs and Ferrostatin-1 [43-44].<br>**Photoreceptors** Limited evidence. mGPx4 and Ferrostatin-1 can regulate ROS accumulation and iron deposition in photoreceptors [49].<br>**RCECs** Cultured RCECs undergo ferroptosis under high glucose stimulation [52]. Inhibition of RCEC ferroptosis improves vascular hyperpermeability [54-55].<br>**RGCs** Direct evidence is lacking. I/R injury induces ferroptosis in RGCs [59].
Pyroptosis	Cookson et al, 2001 [61]	Caspase-1<br>NLRP3<br>ASC<br>GSDMD<br>GSDME<br>MEK7	Inflammation<br>Oxidative stress<br>I/R injury	**RPE** High glucose levels induce pyroptosis in cultured RPE cells and exacerbate inflammation [6-7, 79]. Regulated by non-coding RNAs [6-7, 79-83].<br>**RGCs** Limited research. Genes related to RGC pyroptosis have been identified in animals [85].<br>**Glia** High glucose levels induce pyroptosis in microglia and Müller cells in vitro, which may serve as important pathways glia-mediated inflammation [89-90].<br>**RCECs/pericytes** In vitro evidence. Regulated by non-coding RNAs and NLRP3 [92-99].
Necroptosis	Degterev et al, 2005 [100]	RIPK1<br>RIPK3<br>MLKL	Inflammation<br>Oxidative stress<br>I/R injury	**RGC** Under high glucose stimulation in vitro and in animal models of I/R, RGCs undergo RIPK1/RIPK3/MLKL-dependent necroptosis [115-116].<br>**Microglia** In DR animal, microglia experience RIPK3-dependent necroptosis, exacerbating neuroinflammation and neurodegeneration [122].<br>**Photoreceptors** Limited direct evidence. In retinal detachment models, RIPK3 mediates necroptosis of photoreceptors leading to neurodegeneration [125].
Parthanatos	Dawson et al, 2008 [127]	PARP1<br>AIFM1<br>MIF	Oxidative stress<br>I/R injury	Targeting PARP1-mediated parthanatos holds the potential to promote cell survival and neurovascular protection. Erythropoietin may alleviate DRN by regulating PAR polymer-associated parthanatos [133].
PANoptosis	Malireddi et al 2019 [135]	PANoptosome	I/R injury	Elevation of PANoptosome components and PANoptosis-like cell death are observed in the retinal I/R injury model [138]. Further evidence is required.

Abbreviations: DRN: diabetic retinal neurodegeneration; PCD: programmed cell death; GPX4: glutathione peroxidase 4; GSH: glutathione; PUFA: polyunsaturated fatty acid; ACSL4: acyl-CoA synthetase long chain family member 4; LPCAT3: lysophosphatidylcholine acyltransferase 3; I/R: ischemia-reperfusion; RPE: retinal pigment epithelium; mGPx4: mitochondrial isoform of glutathione peroxidase 4; ROS: reactive oxygen species; RCEC: retinal capillary endothelial cells; RGC: retinal ganglion cell; NLRP3: nod-like receptor family pyrin domain containing 3; ASC: adaptor protein-apoptosis-associated speck-like protein containing a CARD; GSDMD: gasdermin D; GSDME: gasdermin E; MEK7: mitogen-activated protein kinase extracellular signal-regulated kinase 7; RIPK1: receptor-interacting protein kinase 1; RIPK3: receptor-interacting protein kinase 3; MLKL: mixed lineage kinase-like protein; DR: diabetic retinopathy; PARP1: poly (ADP-ribose) polymerase-1; AIFM1: apoptosis inducing factor mitochondria associated 1; MIF: macrophage migration inhibitory factor. PAR: poly (ADP-ribose).
